# Supplementary material for: Anthropogenically-driven increases in the risks of summertime compound hot extremes
Source: Nat Commun. 2020 Feb 11;11:528. doi: 10.1038/s41467-019-14233-8 (PMC7012878; doi:10.1038/s41467-019-14233-8)
Supplement: Supplementary file 1 — Supplementary Information [file 41467_2019_14233_MOESM1_ESM.pdf]

Supplementary Information for

Anthropogenically-driven increases in the risks of summertime compound hot extremes

Wang et al.

Supplementary Notes

Supplementary Note 1 Roles of changing parameters of daily temperature distributions on the occurrence probability of summertime compound hot extremes

We use the generalized extreme value (GEV) distribution to fit the summer daily temperature probability distribution functions (PDFs). The GEV distribution is specified by three parameters: location, scale and shape, which measure the central tendency, dispersion around the central tendency, and skew of PDFs, respectively. They are closely associated with three moments: mean, variance, and skewness, respectively. Specific to the PDFs of summertime daily temperatures, changes in the location parameter correspond to mean-state shifts—general warming signals, while changes in scale and shape parameters are indicative of evolutionary temperature variability. In this study, we employ the GEV fit to illustrate and compare respective roles of general warming and evolving temperature variability on the occurrence probability of summertime compound hot extremes. This analysis serves as a cross-validation for the method presented in the main text.

The GEV distribution framed by location parameter μ, scale parameter σ, and shape parameter $k \neq 0$ is expressed as follows:

$$y = f(x \mid k, u, \sigma) = (\frac{1}{\sigma})\exp(-(1+k\frac{(x-u)}{\sigma})^{-1/k})(1+k\frac{(x-u)}{\sigma})^{-1-1/k}$$

when $1+k\frac{(x-u)}{\sigma} > 0$

The condition of $k = 0$ corresponds to the Type I case (Gumbel).

$k > 0$ corresponds to the Type II case (Fréchet), while $k < 0$ corresponds to the Type III case (Weibull).

We split the historical period of 1960–2012 into two sub-periods (1960–1985 and 1986–2012). The

GEV fit is performed with respect to the former and the latter sub-periods, with fitted PDFs referred to as $PDF_0=f(\mu_0, \sigma_0, k_0)$ and $PDF_1= f(\mu_1, \sigma_1, k_1)$, respectively. For daily maximum/minimum temperatures in each grid-box, we keep the scale and shape parameters constant but shift the location parameter μ from μ_0 to μ_1 (i.e., $PDF_\mu = f(\mu_1, \sigma_0, k_0)$). We then obtain occurrence probabilities of hot days and nights in these two sub-periods by calculating proportions of threshold-exceeding (90th percentile) days within PDF_0 and PDF_μ. By the derived probabilities, we use the bootstrapping technique to generate 100000 random samples of hot days and nights for two sub-periods, and then count the number of days registering hot days and nights simultaneously, i.e., the PDF-fitted frequency of compound hot extremes. The ratio of the difference between these PDF-fitted frequencies during two sub-periods in their observed counterpart is interpreted as the contribution from changing location parameter alone. Similarly, we quantify relative contributions from changing scale parameter alone (i.e., $PDF_\sigma = f(\mu_0, \sigma_1, k_0)$ vs. PDF_0), changing shape parameter alone (i.e., $PDF_k = f(\mu_0, \sigma_0, k_1)$ vs. PDF_0) and changing them both (i.e., $PDF_{\sigma, k} = f(\mu_0, \sigma_1, k_1)$ vs. PDF_0).

Supplementary Note 2 Trends in dynamical conditions and its relationship with the frequency change of compound hot extremes

Both internal variability and anthropogenic warming may result in changes in atmospheric circulation patterns (refs. 29-31 in the main text). To account for both, we calculate trends for both sea level pressure (SLP) and 500hPa geopotential height (HGT) from the NCEP/NCAR R1 Reanalysis (National Centers for Environmental Prediction/National Center for Atmospheric Research)[1] to generally represent regional changes in anticyclonic conditions, as recommended by ref. 29 in the main text.

We first apply a bilinear interpolation to re-grid the reanalysis data onto the HadGHCND's grids (3.75°×2.5°) and mask the re-gridded data by the HadGNCHD data availability. As we focus our analysis on summertime (June-August for the Northern Hemisphere), we calculate the summer-mean time series of SLP and HGT for each grid during 1960–2012. We note that the incorporation of satellite observations since the end of 1978 may result in a spurious jump (inhomogeneity) in the climatology of NCEP-NCAR reanalysis, thereby causing artificial long-term trends for SLP and HGT[2]. To overcome this, following the method developed by the data producer (see refs.2 and 3 below), we divide the trend estimate for the SLP and HGT into two essentially homogenous sub-periods (i.e., pre-satellite period: 1960–1978 and post-satellite period 1979–2012), by using the nonparametric Theil-Sen slope estimator. Weighting these two sub-period trends by the length of time spans (i.e., the former period: 19/53; the latter period: 34/53), we obtain the whole-period trend.

To better serve our purpose of explaining the spatial heterogeneity of trends for compound hot extremes, we divide the Northern Hemisphere continents into twenty nearly equal-area climate

zones basically following the classification scheme proposed by ref. 4. The geographical realms of them are shown in Fig. 3a in the main text, with their acronyms and boundaries detailed in Supplementary Table 2. On this basis, we calculate regional-average trends for frequency of compound hot extremes, sea level pressure, and 500hPa geopotential height. We then use the ordinary least squares regression to quantify the statistical relationship between trends for dynamic conditions and frequency of compound hot extremes. We also compute the Pearson correlation coefficient between two variables and its corresponding *p*-values for the two-tailed test to determine its statistical significance, as presented in Fig. 3b-e in the main text.

Supplementary Note 3 Detection and attribution of observed changes in independent hot extremes

For summertime independent hot days, the simulated (multi-model ensemble–MME mean) frequency and intensity trends are slightly stronger than observed (Supplementary Fig. 9a, b); while the simulated (MME mean) frequency and intensity changes in summertime independent hot nights are markedly weaker than observed (Supplementary Fig. 9c, d). This seems to be partly associated with underestimation of the decreasing trend of diurnal temperature range in CMIP5 climate models[5], which would have induced biased warming (cooling) trend in Tmax (Tmin), thus overestimating (underestimating) the frequency and intensity changes in independent hot days (nights).

Both the anthropogenic forcing (ANT) and natural forcing (NAT) signals can be detected in changes of independent hot days and nights (Supplementary Fig. 10). The simulations tend to slightly overestimate (markedly underestimate) the human-induced frequency and intensity changes in independent hot days (nights). This agrees with previous studies on conventional univariate-based temperature extremes, which reported that CMIP5 models overestimated the frequency change of warm days, particularly in summer[6]. Such overestimation may artificially accelerate the transition of independent hot nights to compound hot extremes in summer in simulations (Supplementary Fig. 11e and Supplementary Fig. 12e). In the three-signal analysis, other anthropogenic forcings (OANT; i.e., anthropogenic aerosols and large-scale land use changes) fail to be detected in independent hot days' changes, while could be detected in changes of independent hot nights with relatively large uncertainties. This difference may be ascribed to misrepresentations of the diurnal variations of aerosols' influences and/or other forcings in models (e.g., the poorly-represented indirect effects

of aerosols on clouds and precipitation). Amongst these three external forcers, the rise in greenhouse gases (GHG) is found to be the most dominant contributor to the frequency and intensity changes in independent hot days and nights, with a small offset from OANT forcings and negligible impacts from NAT forcings.

Supplementary Tables

Supplementary Table 1 The CMIP5 models used in the detection and attribution analysis. Listed are the ensemble size of the ALL-forcing, NAT-forcing, GHG-forcing experiments, the chunks of pre-industrial (pi) control simulations, and the horizontal resolution of climate models.

Model	ALL ensemble size	NAT ensemble size	GHG ensemble size	pi-control length (chunks)	Horizontal resolution (latitude & longitude)		Length of Calendar Year (days)
CanESM2	5	5	5	18	2.7906°	2.8125°	365
CNRM-CM5	6	6	6	10	1.4008°	1.40625°	365/366
CSIRO-Mk3-6-0	10	10	10	8	1.8653°	1.875°	365
HadGEM2-ES	4	4	4	10	1.25°	1.875°	360
IPSL-CM5A-LR	3	3	3	18	1.8947°	3.75°	365
total	28	28	28	64			

Supplementary Table 2 The geographical boundaries of climate zones

Region name	Acronym	Latitude (°)	Longitude (°)
Alaska	ALA	60N-72N	170W-103W
Northwestern North America	NWNA	50N-60N	130W-103W
Southwestern North America	SWNA	30N-50N	130W-103W
Central North America	CNA	30N-50N	103W-85W
Eastern North America	ENA	30N-50N	85W-60W
Northern North America	NNA	50N-70N	103W-85W
Greenland	GRL	50N-85N	85W-10W
Northwestern Europe	NWEU	48N-75N	10W-22E
Northeastern Europe	NEEU	48N-75N	22E-40E
Mediterranean Basin	MED	30N-48N	10W-40E
Western part of Central Asia	WCAS	30N-50N	40E-60E
Eastern part of Central Asia	ECAS	30N-50N	60E-75E
Northwestern Asia	NWAS	50N-70N	40E-75E
Western Siberia	WSBR	50N-70N	75E-100E
Central Siberia	CSBR	50N-70N	100E-125E
Eastern Siberia	ESBR	50N-70N	125E-180E
Tibet	TIB	30N-50N	75E-100E
Northeastern Asia1 (Central-North China)	NEAS1	30N-50N	100E-125E
Northeastern Asia2 (Northeastern China, Korea,	NEAS2	30N-50N	125E-145E

Japan)			
Southeastern Asia	SEAS	10N-30N	100E-125E

Supplementary Figures

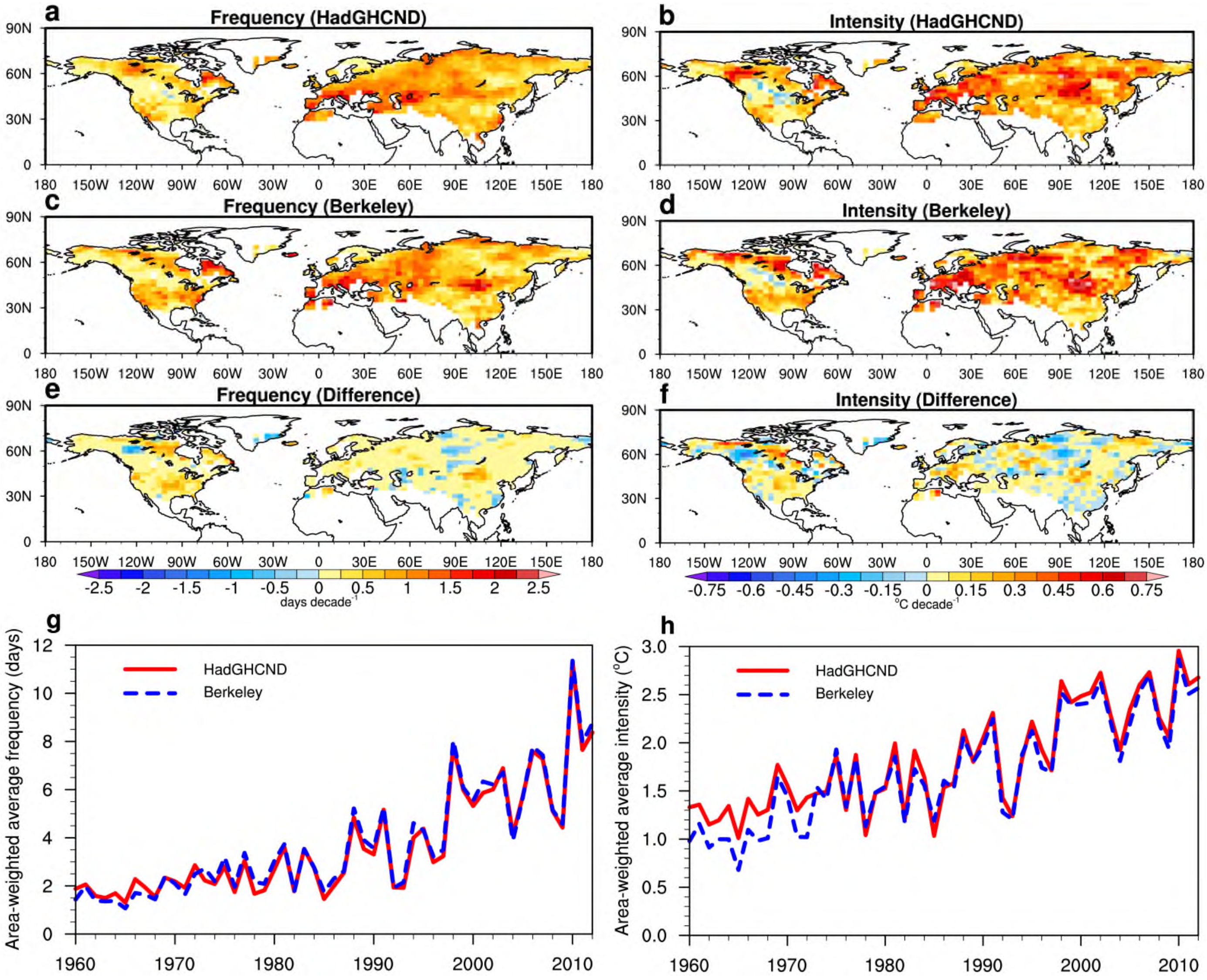

Supplementary Figure 1 Robustness test for trends' dependence on datasets. Linear trends are estimated for frequency and intensity of summertime compound hot extremes for the period of 1960–2012 using the HadGHCND observations (**a**, **b**), the Berkeley Earth Surface Temperature data set (**c**, **d**), and their differences (**e**, **f**, Berkeley minus HadGHCND). **g**, **h** show area-weighted mean frequency (**g**) and intensity (**h**) of summertime compound hot extremes across the Northern Hemisphere lands during 1960–2012.

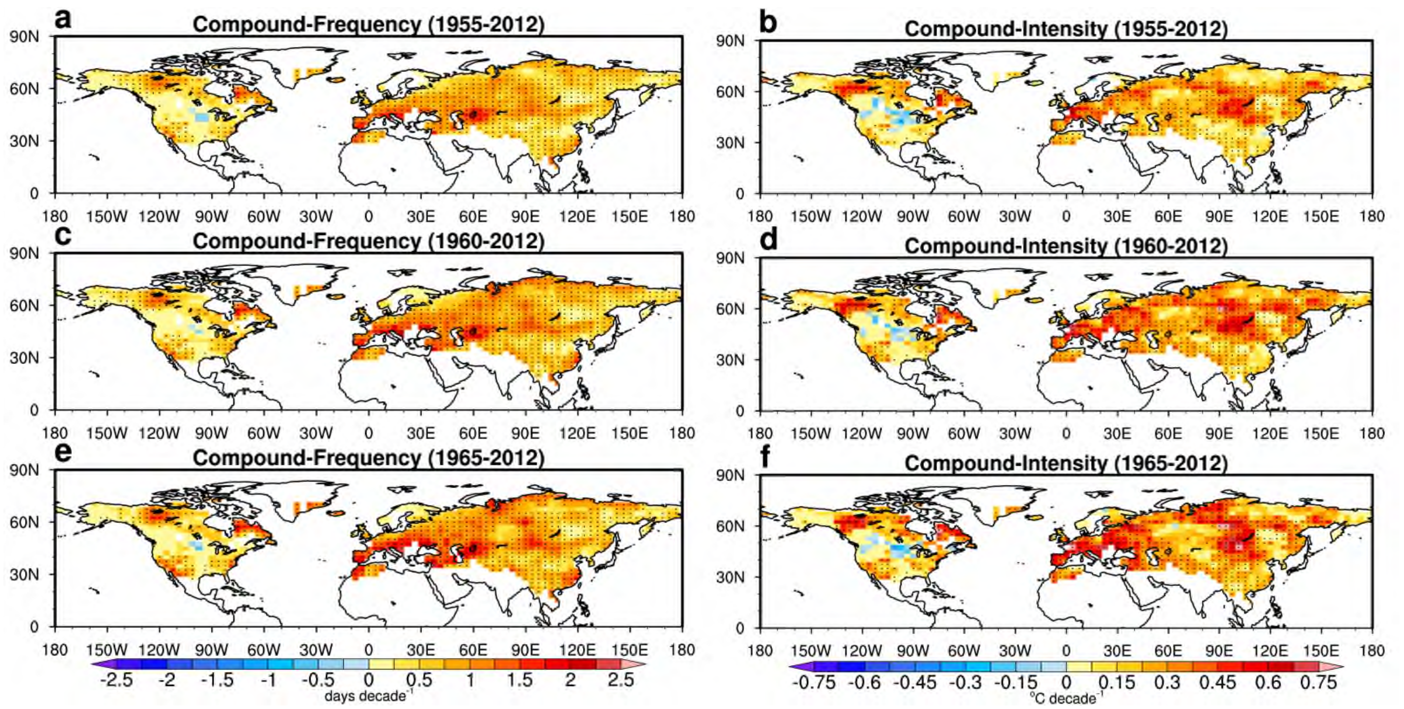

Supplementary Figure 2 Robustness test for trends' dependence on analysis periods. Linear trends for frequency and intensity of summertime compound hot extremes are estimated for the period of 1955–2012 (**a, b**), 1960–2012 (**c, d**), and 1965–2012 (**e, f**) based on the HadGHCND observations. Stipples indicate significance at the 0.05 level.

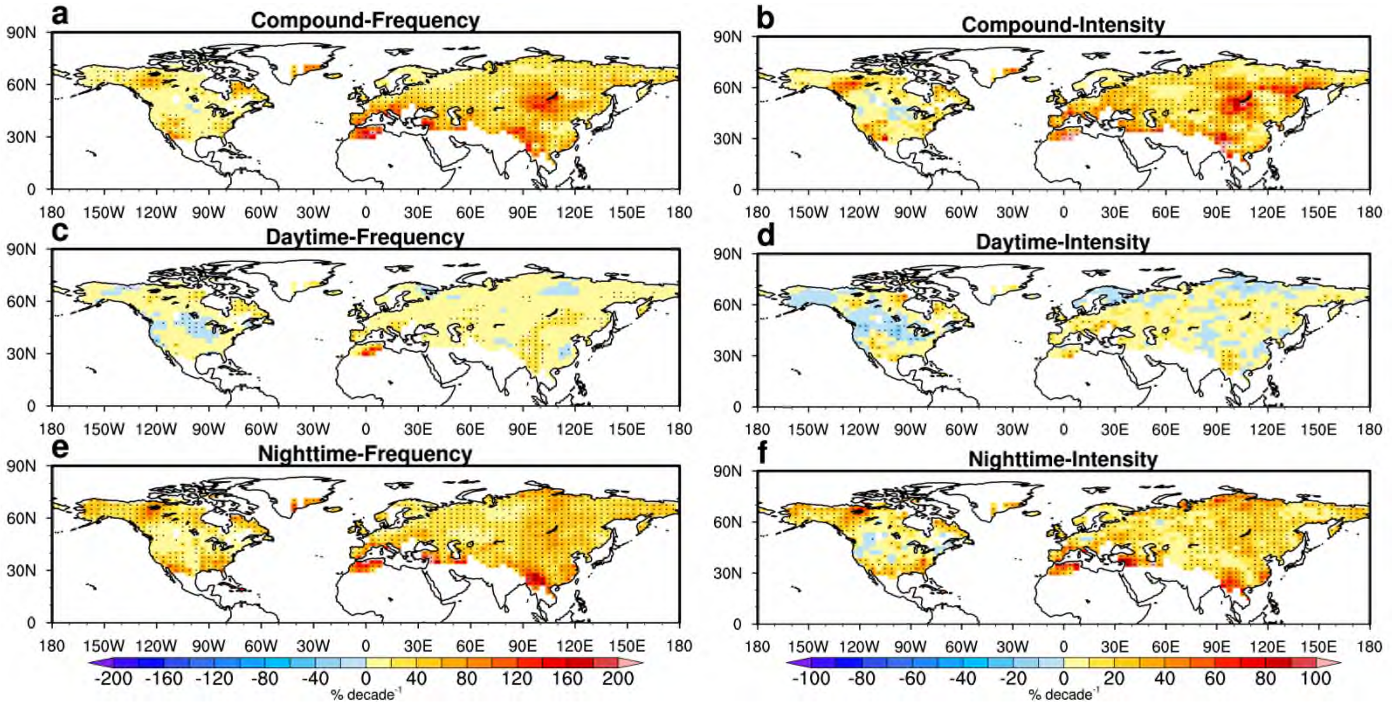

Supplementary Figure 3 **Observed relative changes (% decade^{-1}) in summertime hot extremes.** Linear trends are estimated for frequency and intensity for the period of 1960–2012 based on the HadGHCND observations, with respect to compound hot extremes (**a, b**), independent hot days (**c, d**), and independent hot nights (**e, f**). All trends are converted to percentage changes (% decade^{-1}) relative to their climatology over 1961–1990. Stipples indicate significance at the 0.05 level.

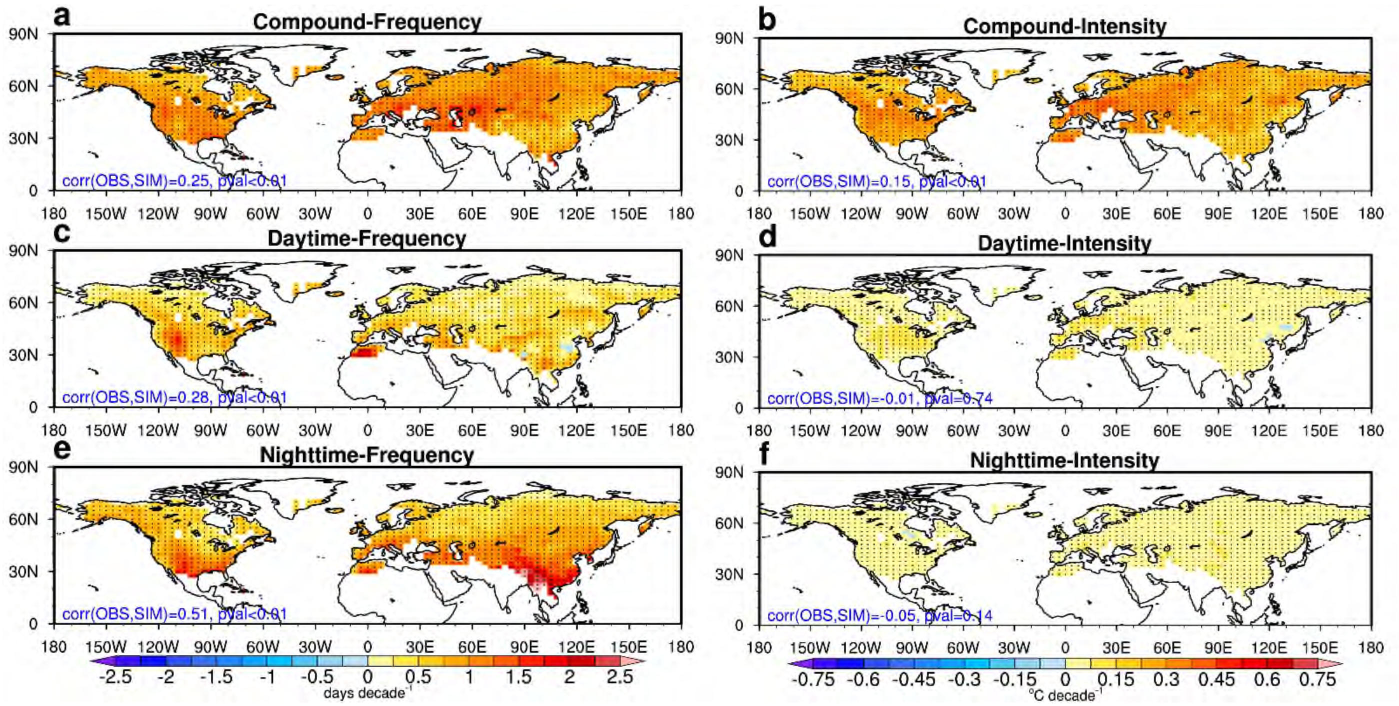

Supplementary Figure 4 **Simulated changes in summertime hot extremes.** Linear trends are estimated for simulated frequency and intensity for the period of 1960–2012, with respect to compound hot extremes (**a, b**), independent hot days (**c, d**), and independent hot nights (**e, f**). Results are based on the multi-model ensemble (MME) means from five CMIP5 models with ALL forcings included. Stipples indicate significance at the 0.05 level. The spatial correlation coefficients between observed and MME mean-simulated trends are marked at the lower-left corner, along with corresponding *p*-values.

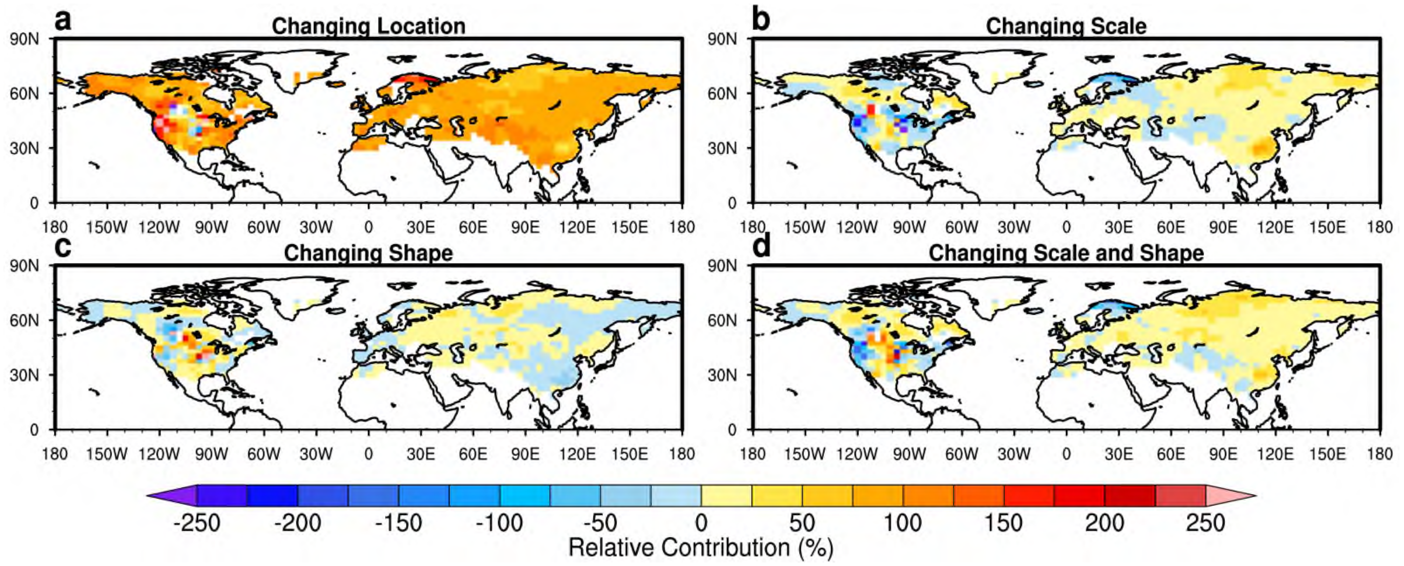

Supplementary Figure 5 Contributions of changes in different distribution parameters. Relative contributions from changing location parameter alone (**a**), scale parameter alone (**b**), shape parameter alone (**c**), and changing scale and shape parameters simultaneously (**d**) to observed frequency changes in summertime compound hot extremes during 1960–2012. Methods are detailed in Supplementary Note 1.

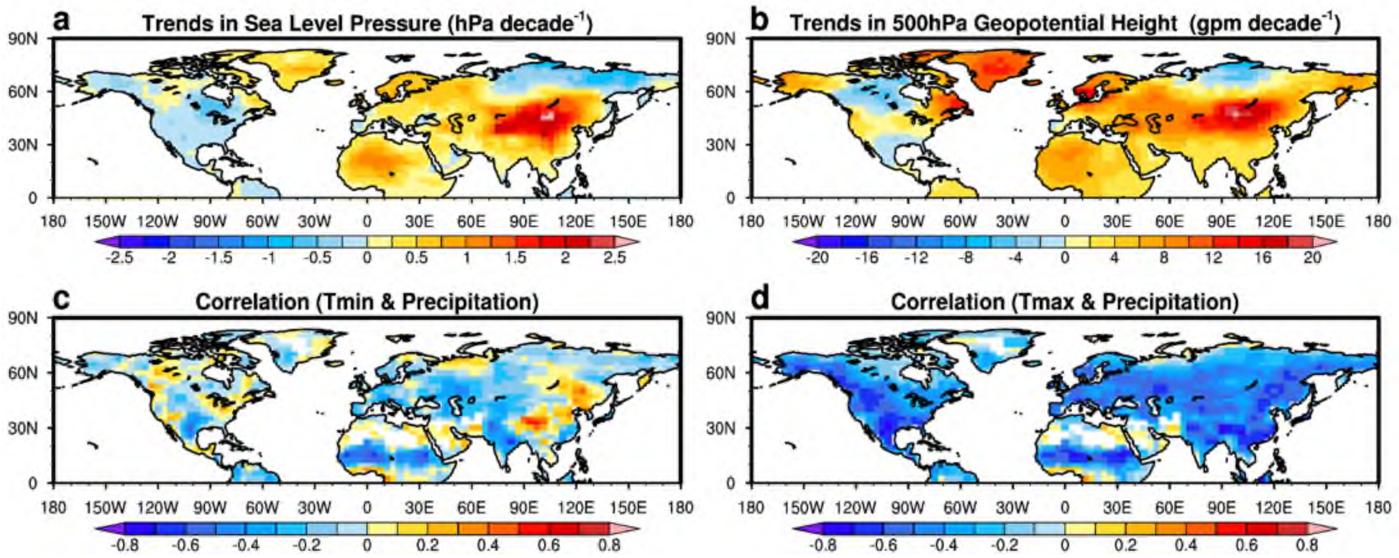

Supplementary Figure 6 Dynamic-thermodynamic processes influencing trend patterns. Presented include linear trends for summer-mean sea level pressure (**a**) and 500hPa geopotential height (**b**) based on the NCEP-NCAR R1 reanalysis, and correlation coefficients between CRU summertime monthly-mean daily maximum (**c**) /minimum temperature (**d**) and precipitation during 1960–2012. For the method for trend estimate in (**a**) and (**b**) see Supplementary Note 2. Before calculating correlation coefficients in (**c**) and (**d**), both monthly-mean maximum/minimum temperature and precipitation at each grid are linearly detrended.

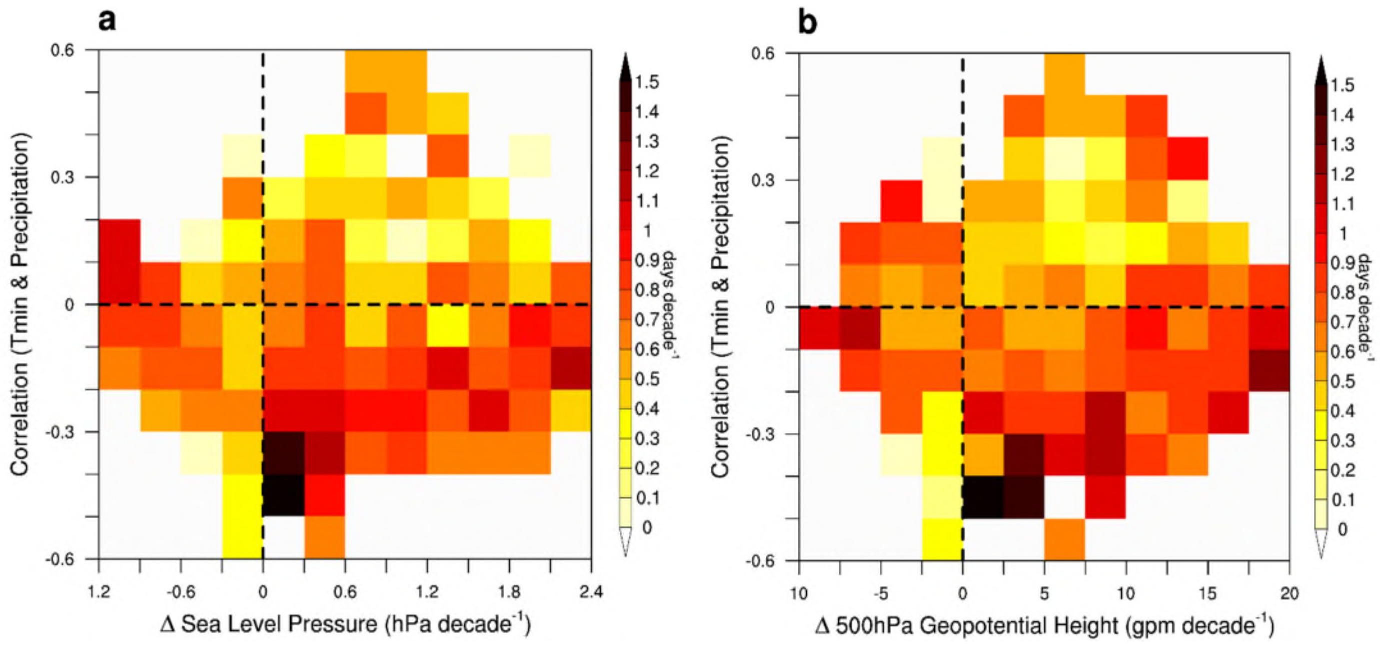

Supplementary Figure 7 Joint influences of dynamic-thermodynamic drivers on trend patterns. Observed trends for sea level pressure (**a**; SLP, x-axis), 500hPa geopotential height (**b**; HGT, x-axis) and correlations between summertime monthly-mean daily minimum temperature and precipitation (CORR, y-axis) are binned into intervals of 0.3 hPa decade^{-1}, 2.5gpm decade^{-1} and 0.1 (CORR), respectively. The color of each square indicates the mean frequency trend averaged amongst those grids with their SLP/HGT trends and CORRs falling into corresponding bins. This is a synthesis plot for those one-dimensional plots as presented in **Fig. 3** in the main text.

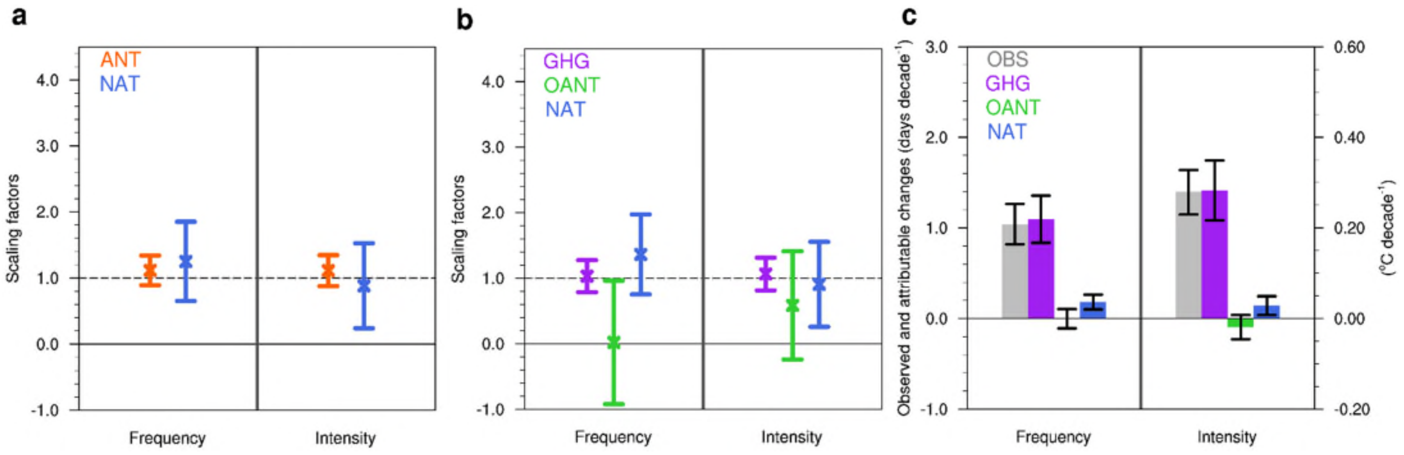

Supplementary Figure 8 Scaling factors and attributable changes for compound hot extremes. **a** The best estimate (cross) and 5%–95% uncertainty range (bar) of scaling factors for ANT (anthropogenic, orange) and NAT (natural, blue) forcings. **b** Same as **a** but for GHG (greenhouse gases, purple), OANT (other anthropogenic, green), and NAT (blue) in the three-signal detection analysis. **c** The best estimate (shading) for observed changes (gray) and those attributable to GHG (purple), OANT (green) and NAT (blue), with black bars representing the 90% confidence interval for observed trends and the 5%–95% uncertainty range for attributable trends. The calculations of confidence interval for observed trends and the uncertainty range for attributable changes are detailed in **Methods** in the main text. This figure differs from **Fig. 5** in the main text in using five-year-mean time series instead.

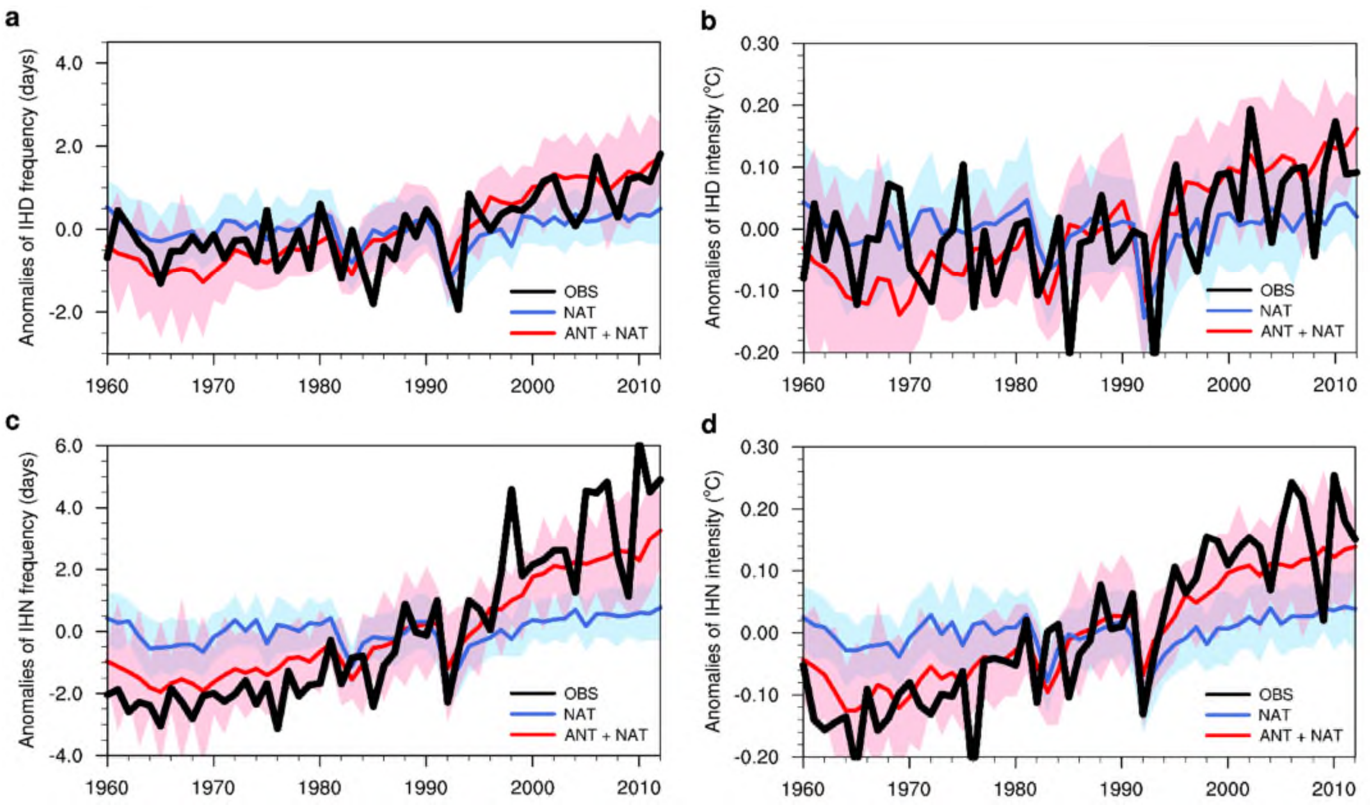

Supplementary Figure 9 **Hemispheric-average indices of independent hot extremes**. **a, b** Anomalies in area-weighted mean frequency (**a**) and intensity (**b**) of independent hot days (IHD) during 1960–2012. **c, d** Same as **a, b** but for independent hot nights (IHN). All anomalies are relative to the 1960–2012 mean. Shown include observations (black line); the MME (multi-model ensemble) mean simulations forced jointly by ANT (anthropogenic) and NAT (natural) forcings (ALL; red line) and the 5%–95% range of ALL responses among individual simulations (red shading); and the MME mean simulations forced only by NAT forcings (blue line) and the 5%–95% range of NAT responses among individual simulations (blue shading).

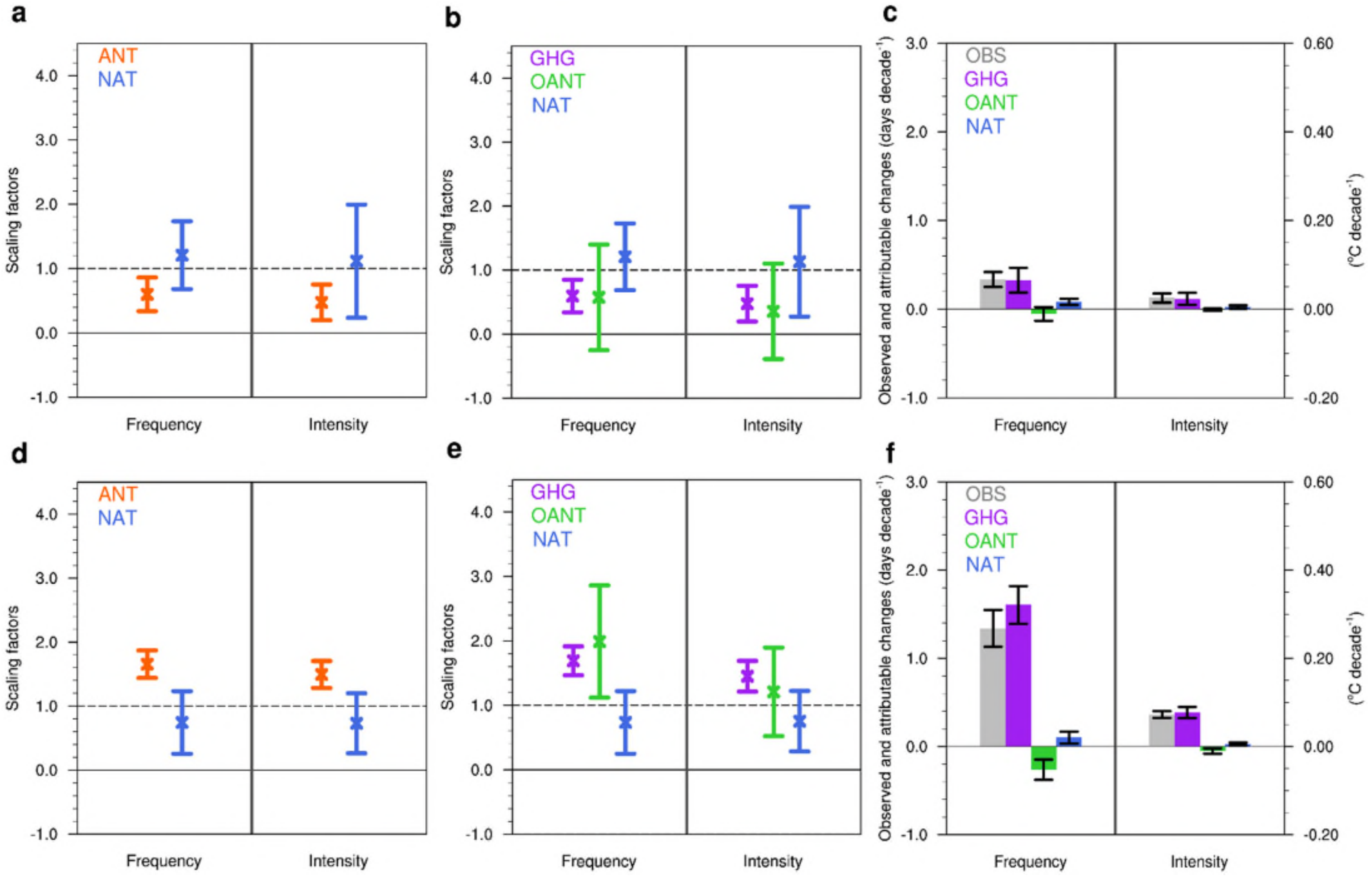

Supplementary Figure 10 **Detection and attribution for independent hot extremes. a** The best estimate (cross) and 5%-95% uncertainty range (bar) of scaling factors for ANT (anthropogenic, orange) and NAT (natural, blue) forcings for independent hot days. **b** Same as **a** but for GHG (greenhouse gases, purple), OANT (other anthropogenic, green), and NAT (blue) in the three-signal detection analysis. **c** The best estimate (shading) for observed changes (gray) and those attributable to GHG (purple), OANT (green) and NAT (blue), with black bars representing the 90% confidence interval for observed trends and the 5%–95% uncertainty range for attributable trends. **d-f** Same as **a-c**, but for independent hot nights. The calculations of confidence interval for observed trends and the uncertainty range for attributable changes are detailed in **Methods** in the main text.

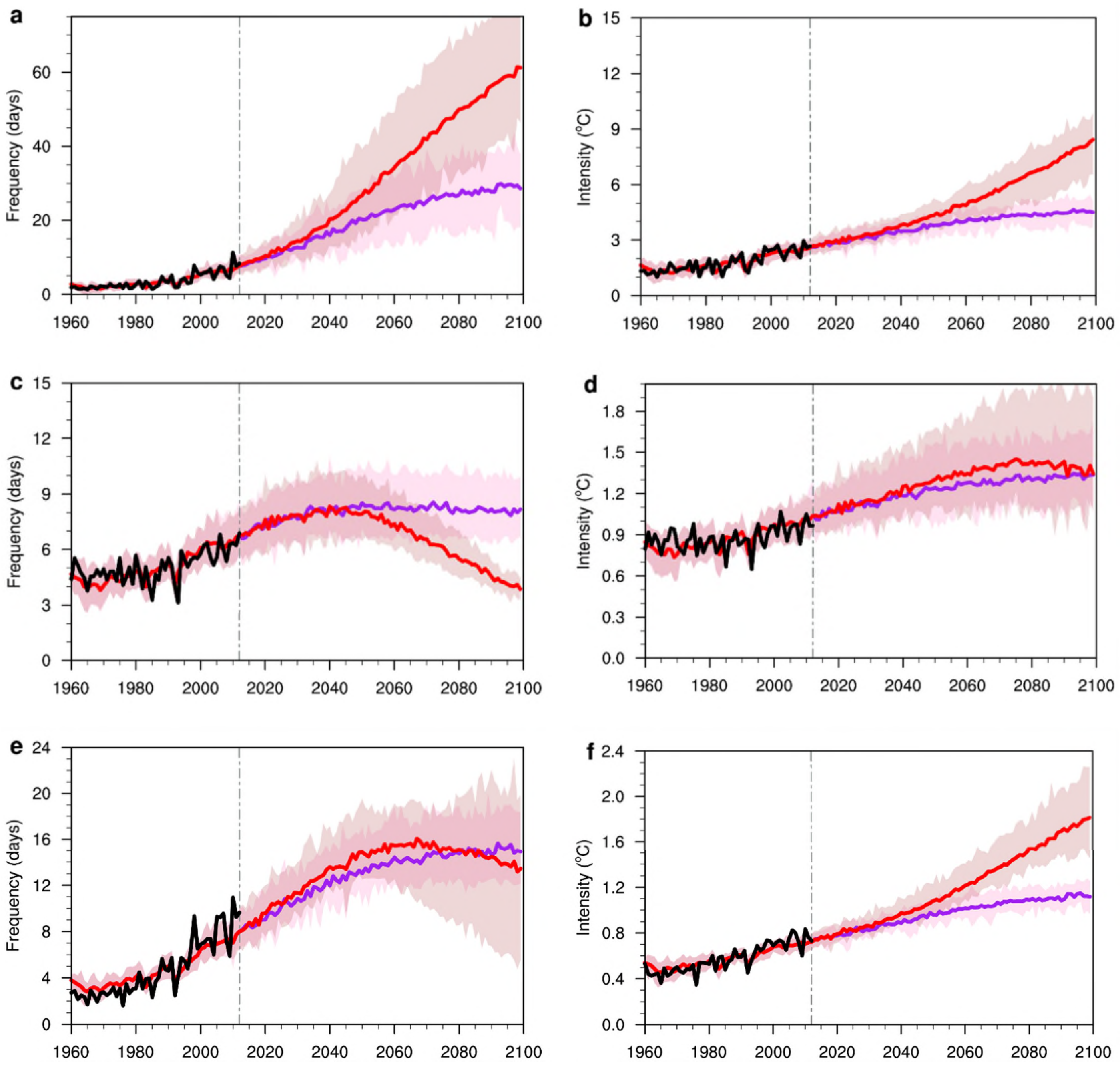

Supplementary Figure 11 Raw simulations and projections of summertime hot extremes. Area-weighted hemispheric-average frequency and intensity of compound hot extremes (**a**, **b**), independent hot days (**c**, **d**), and independent hot nights (**e**, **f**). Shown are observational series (black curve), the MME (multi-model ensemble) mean historical series and future projections under RCP4.5 (purple curve) and RCP8.5 (red curve). Shadings in corresponding colors enclose the

5%–95% range of individual simulations for each type. The dashed vertical line indicates the start year of projections, i.e., 2013.

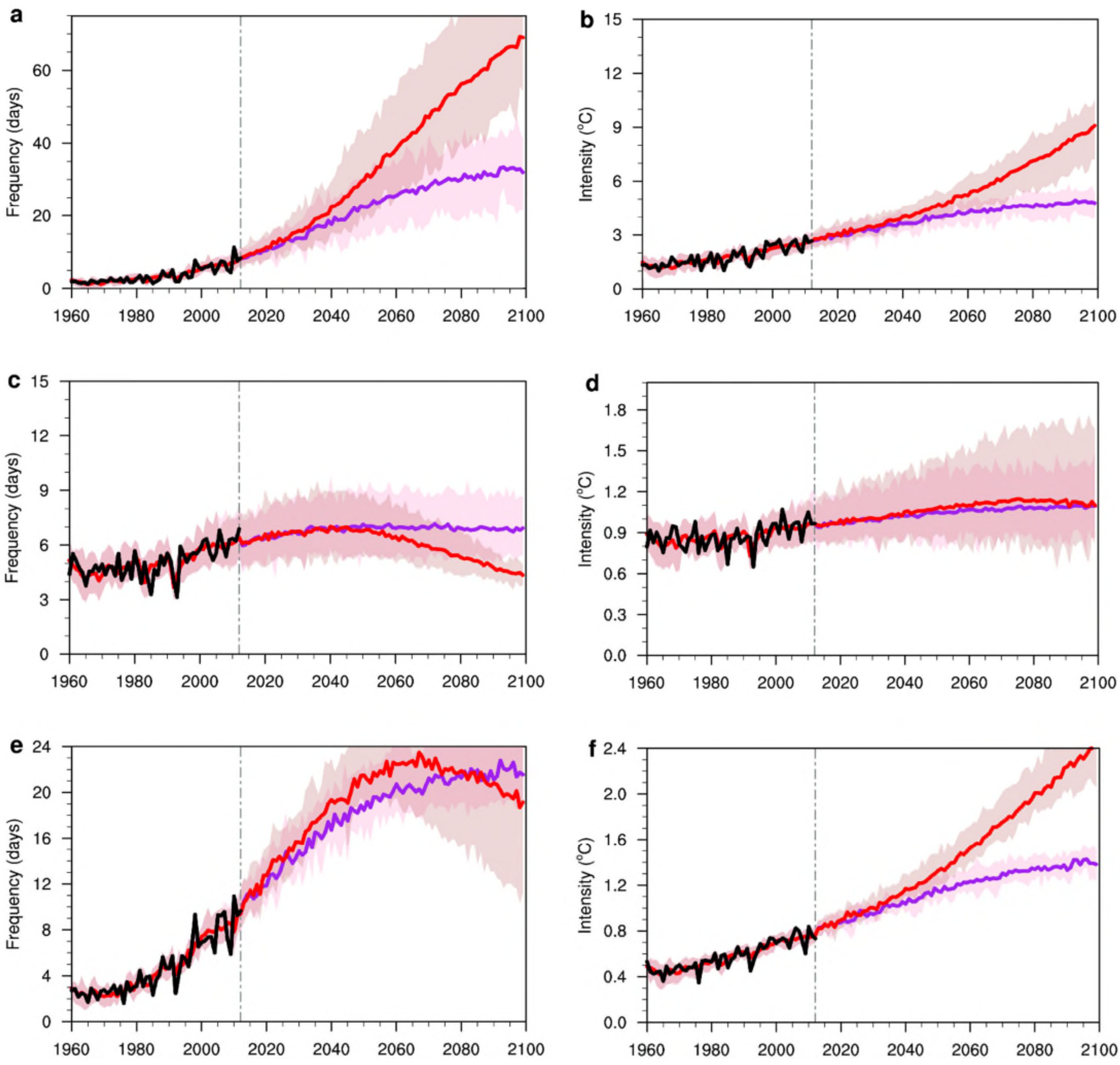

Supplementary Figure 12 **Constrained simulations and projections of summertime hot extremes.** Area-weighted hemispheric-average frequency and intensity of compound hot extremes(**a, b**), independent hot days (**c, d**), and independent hot nights (**e, f**). Shown are observational series (black curve), the MME (multi-model ensemble) mean historical series and future projections under RCP4.5 (purple curve) and RCP8.5 (red curve). Shadings in

corresponding colors enclose the 5%–95% range of individual simulations for each type. The dashed vertical line indicates the start year of projections, i.e., 2013. This figure is a supplementary to **Fig. 6** in the main text by overlaying observational series to highlight the calibration effect. See **Methods** in the main text for the observationally-constrained projection technique.

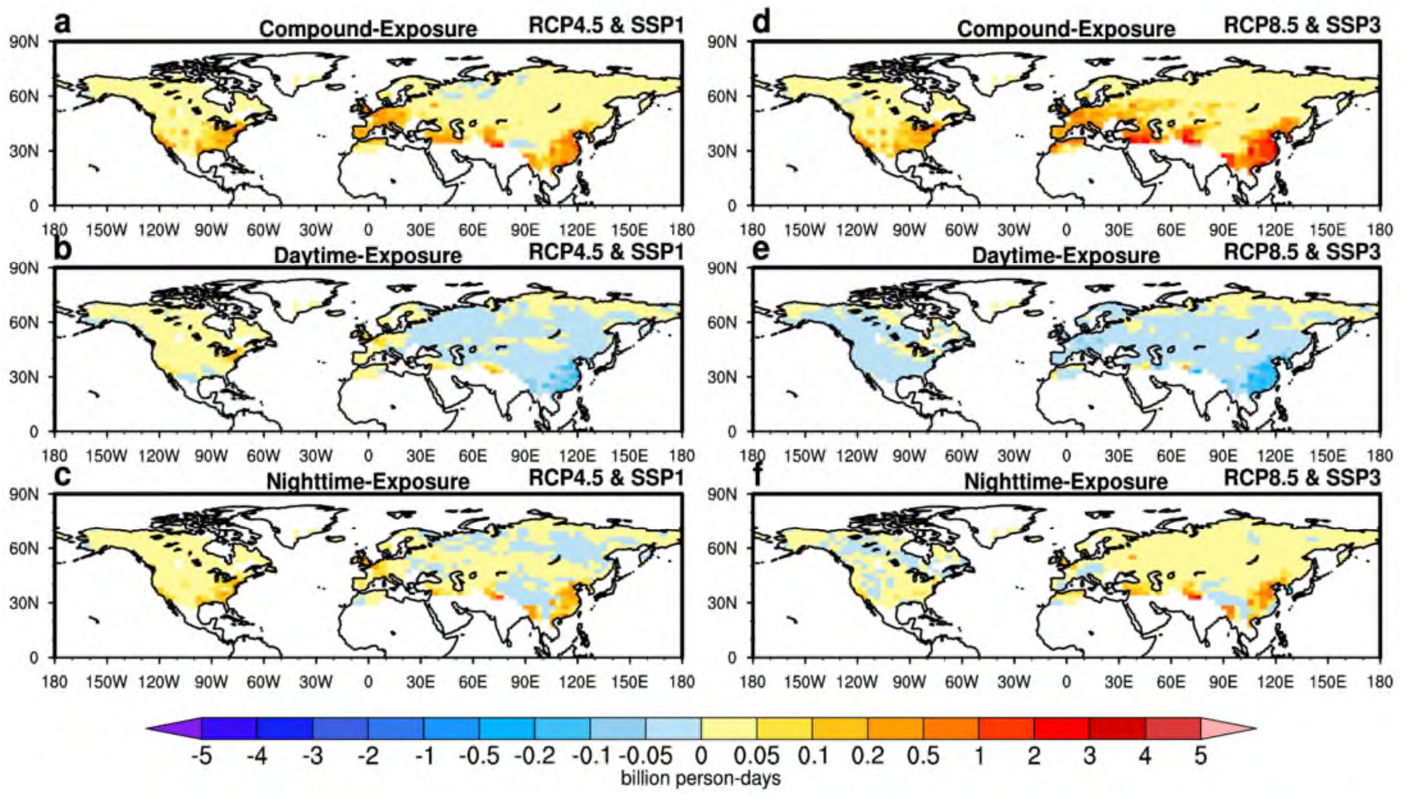

Supplementary Figure 13 **Spatial patterns for projected changes in population exposure.** Changes in exposure to summertime hot extremes are measured by the differential exposure between the decades of 2010s and 2090s (the 2090s minus the 2010s). **a**, **b** Exposure to compound hot extremes; **c**, **d** Exposure to independent hot days; **e**, **f** Exposure to independent hot nights. Shown are MME (multi-model ensemble) mean projected changes in two integrated scenarios designed as RCP4.5 (climate)-SSP1 (population) combination in **a**-**c**, and RCP8.5 (climate)-SSP3 (population) combination in **d**-**f**.

Supplementary References

1. Kalnay, E. et al. The NCEP/NCAR 40-year reanalysis project. *Bull. Amer. Meteorol. Soc.* **77**, 437–472 (1996).

2. Kalnay, E., Cai, M., Li, H. & Tobin, J. Estimation of the impact of land-surface forcings on temperature trends in eastern United States. *J. Geophys. Res. Atmos.* **111**, D06106 (2006).

3. Kalnay, E. & Cai, M. Impact of urbanization and land-use change on climate. *Nature* **423**, 528–531 (2003).

4. Giorgi, F. & Francisco, R. Uncertainties in regional climate change prediction: a regional analysis of ensemble simulations with the HADCM2 coupled AOGCM. *Clim. Dyn.* **16**, 169–182 (2000).

5. Lewis, S. C. & Karoly, D. J. Evaluation of historical diurnal temperature range trends in CMIP5 Models. *J. Clim*. **26**, 9077–9089 (2013).

6. Morak, S., Hegerl, G. C. & Christidis, N. Detectable changes in the frequency of temperature extremes. *J. Clim.* **26**, 1561–1574 (2013).
